# Functional Properties of the Catalytic Domain of Mouse Acidic Mammalian Chitinase Expressed in *Escherichia coli*

**DOI:** 10.3390/ijms16024028

**Published:** 2015-02-13

**Authors:** Akinori Kashimura, Masahiro Kimura, Kazuaki Okawa, Hirotaka Suzuki, Atsushi Ukita, Satoshi Wakita, Kana Okazaki, Misa Ohno, Peter O. Bauer, Masayoshi Sakaguchi, Yasusato Sugahara, Fumitaka Oyama

**Affiliations:** 1 Department of Applied Chemistry, Kogakuin University, Hachioji, Tokyo 192-0015, Japan; E-Mails: bd12001@ns.kogakuin.ac.jp (A.K.); b411050@ns.kogakuin.ac.jp (M.K.); bd14001@ns.kogakuin.ac.jp (K.O.); hirotaka.suzuki@ssd.hpk.co.jp (H.S.); atsushi.ukita@gmail.com (A.U.); bm13028@ns.kogakuin.ac.jp (S.W.); kanaokazaki@eurofins.com (K.O.); bd13001@ns.kogakuin.ac.jp (M.O.); bt11532@ns.kogakuin.ac.jp (M.S.); bt79310@ns.kogakuin.ac.jp (Y.S.); 2 Department of Neuroscience, Mayo Clinic, Jacksonville, FL 32224, USA; E-Mail: Bauer.Peter@mayo.edu

**Keywords:** acidic mammalian chitinase, allergy, asthma, catalytic domain, chitin-binding activity, chitinolytic activity, colloidal and crystalline chitin, food processing, innate immunity, mouse

## Abstract

Mouse acidic mammalian chitinase (AMCase) plays important physiological roles in defense and nutrition. AMCase is composed of an *N*-terminal catalytic domain (CatD) and a *C*-terminal chitin-binding domain (CBD). We expressed CatD of mouse AMCase as a recombinant fusion protein with Protein A and V5-His in *Escherichia coli* (Protein A-CatD-V5-His), evaluated its functional properties and compared them to the full-length AMCase (Protein A-AMCase-V5-His). Under our experimental conditions, the chitinolytic activity of both proteins against 4-nitrophenyl *N,N'*-diacetyl-β-d-chitobioside was equivalent with regard to their specific enzymatic activities, optimal pH and temperature as well as to the pH and temperature stability. CatD bound to chitin beads and cleaved the *N*-acetylglucosamine hexamer, colloidal and crystalline chitin as well as the shrimp shell, and released primarily *N,N'*-diacetylchitobiose fragments at pH 2.0. These results indicate that the primary structure of CatD is sufficient to form a proper tertiary structure required for chitinolytic activity, recognize chitin substrates and degrade them in the absence of a CBD. Our recombinant proteins can be used for further studies evaluating pathophysiological roles of AMCase in different diseases.

## 1. Introduction

Chitinases are enzymes that digest chitin, a polymer of (β-1,4)-linked *N*-acetyl-d-glucosamine (GlcNAc). Chitin is an integral component of the exoskeletons of crustaceans and insects, and the microfilarial sheaths of parasitic nematodes and fungal cell walls [1,2]. Chitinases are produced by various organisms, including bacteria, fungi, plants, nematodes, and arthropods [1,2,3] and are thought to have important roles in chitin digestion for organism defense or for providing a source of carbon and energy [4].

Despite the absence of endogenous chitin, humans and mice express two active chitinases, designated as chitotriosidase (Chit1) and acidic mammalian chitinase (AMCase). Both enzymes show sequence homology to bacterial chitinases and belong to the family 18 of glycoside hydrolases [3,5,6]. Chit1 was the first purified and cloned mammalian chitinase [7,8]. The second mammalian chitinase, AMCase, was named for its acidic isoelectric point [9]. Mouse AMCase has been shown to be most active at pH 2.0 and is acid-stable, whereas Chit1 is inactivated at low pH conditions [9,10].

AMCase has attracted substantial attention due to its increased expression under certain pathological conditions. Significant increase of the AMCase mRNA and protein levels was detected in an induced asthma mouse model [11]. Moreover, AMCase polymorphisms and haplotypes have been reported to be associated with bronchial asthma in humans [12,13] and inhibition of AMCase has been suggested as a therapeutic strategy for this disease [11,14,15]. AMCase expression is also increased in antigen-induced mouse models of allergic lung inflammation [16]. Furthermore, several researchers indicate that AMCase is involved in eye [17,18,19] and stomach diseases [20,21]. However, knowledge on the pathophysiological functions of AMCase in mice and humans has been limited so far.

Recently, we reported that AMCase mRNA is synthesized in mouse stomach at extraordinarily high levels that are comparable to the mRNA level of pepsinogen C (progastricsin), a major component of the gastric mucosa [22,23]. To biochemically characterize the AMCase protein, we established an *Escherichia coli* expression system that allows for the periplasmic production of active AMCase as a recombinant protein fused with Protein A, V5 epitope and (His)_6_ tag (V5-His) (Protein A-AMCase-V5-His) [24]. The *E. coli*-produced AMCase showed a robust peak of activity at pH 2.0, cleaved colloidal chitin and primarily released *N,N'*-diacetylchitobiose fragments, displaying properties similar to those of the protein expressed in CHO cell-expressed [24].

Mouse AMCase is composed of an *N*-terminal catalytic domain (CatD) and a *C*-terminal chitin-binding domain (CBD) [9]. It has been suggested that CBD recognizes chitin and CatD degrades it [25]. However, the functional roles of CatD and CBD of mouse AMCase in the digestion of chitin remains to be elucidated, particularly with regard to the high molecular weight of crystalline chitin and shrimp shell. In this study, we expressed the CatD of mouse AMCase as a recombinant fusion protein of Protein A and V5-His (Protein A-CatD-V5-His) and found that it bound to chitin beads, cleaved GlcNAc hexamer, colloidal and crystalline chitin, and released primarily the *N,N'*-diacetylchitobiose fragments at pH 2.0. Thus, the CatD of mouse AMCase is sufficient for chitin substrates recognition and degradation.

## 2. Results

### 2.1. Recombinant N-Terminal Catalytic Domain (CatD) Expression Plasmids

**Figure 1:**
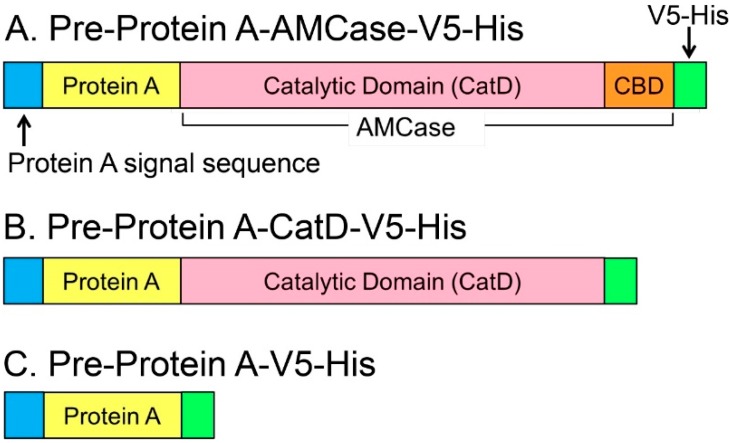
Schematic representations of the *Escherichia coli*-expressed fusion proteins. Mouse AMCase contains an *N*-terminal catalytic domain (CatD) and a *C*-terminal chitin-binding domain (CBD). (**A**) Pre-Protein A-AMCase-V5-His; (**B**) Pre-Protein A-CatD-V5-His; and (**C**) Pre-Protein A-V5-His. The newly synthesized recombinant proteins contain the Protein A signal sequence, which is processed during secretion.

The schematic representations of the recombinant mouse AMCase and CatD are shown in Figure 1. For production in *E. coli*, mature AMCase-V5-His cDNA or its CatD region was cloned into pEZZ18 vector, containing the signal sequence of *Staphylococcus aureus* Protein A to express precursor forms of recombinant proteins of pre-Protein A-AMCase-V5-His and pre-Protein A-CatD-V5-His (Figure 1A,B and Figure S1A). Using a similar approach, we also constructed pre-Protein A-V5-His (Figure 1C).

Expression of these constructs in *E. coli* led to secretion of the mature forms of the recombinant proteins Protein A-AMCase-V5-His, Protein A-CatD-V5-His (Figure S1B) and Protein A-V5-His to the periplasmic space of *E. coli.* These fusion proteins can be purified by IgG Sepharose chromatography or Ni Sepharose chromatography mediated by Protein A or His-tag, respectively (Figure 1). We detected the levels of these recombinant proteins by Western blot using anti-V5 epitope antibody and measured their chitinolytic activity using 4-nitrophenyl *N,N′*-diacetyl-β-d-chitobioside as a substrate.

### 2.2. Purification of the Recombinant CatD from the Periplasmic Fraction of Escherichia coli

**Figure 2:**
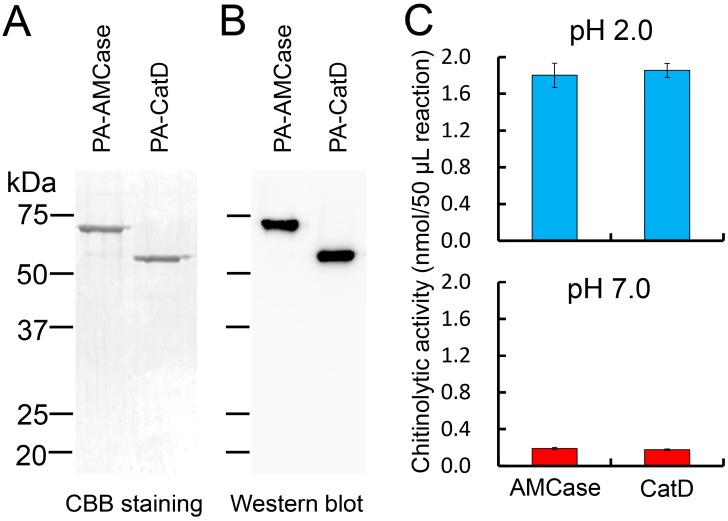
Analysis of the *E. coli*-expressed fusion proteins. (**A**) 12.5% SDS-PAGE analysis of the recombinant proteins from the periplasmic fractions (Peri 1) of *E. coli*. The fusion proteins were purified by IgG Sepharose followed by Ni Sepharose chromatography, electrophoresed (1 μg of protein) and visualized in the gel by Coomassie Brilliant Blue R-250 (CBB); (**B**) Western blot analysis of the recombinant proteins. 0.1 μg protein separated by SDS-PAGE was transferred to a PVDF membrane. Immunoblot was performed with anti-V5-HRP antibody. PA-AMCase, Protein A-AMCase-V5-His; PA-CatD, Protein A-CatD-V5-His; (**C**) Comparison of the chitinolytic properties of the CatD with the full-length AMCase in 50 μL reactions using 0.1 M Gly-HCl buffer (pH 2.0) or McIlvaine’s buffer (pH 7.0) at 37 °C for 30 min as described in the Experimental Section.

The protein present in the periplasmic fraction 1 (Peri 1) was purified by IgG Sepharose chromatography, followed by Ni Sepharose chromatography. The purified fraction was subjected to SDS-PAGE and then visualized with Coomassie Brilliant Blue R-250 staining (CBB). As shown in Figure 2A, levels of AMCase and CatD were similar. We next evaluated the levels of these proteins by Western blot using anti-V5 antibody, confirming the comparable levels of both recombinant proteins, with signals of AMCase and CatD at approximately 68 and 61 kDa, respectively (Figure 2B), representing the full length AMCase and CatD (without CBD) (Figure 1A,B).

### 2.3. Recombinant CatD Has Chitinolytic Activity

Next, we measured the chitinolytic activity of Protein A-CatD-V5-His at pH 2.0 (Gly-HCl buffer) and pH 7.0 (McIlvaine buffer). It showed a strong chitinolytic activity as measured using 4-nitrophenyl *N,N′*-diacetyl-β-d-chitobioside chromogenic substrate (Figure 2C), which was comparable to that of the full-length AMCase at both pH 2.0 and 7.0 (Figure 2C).

### 2.4. Characterization of the Chitinolytic Activity of Protein A-CatD-V5-His

**Figure 3:**
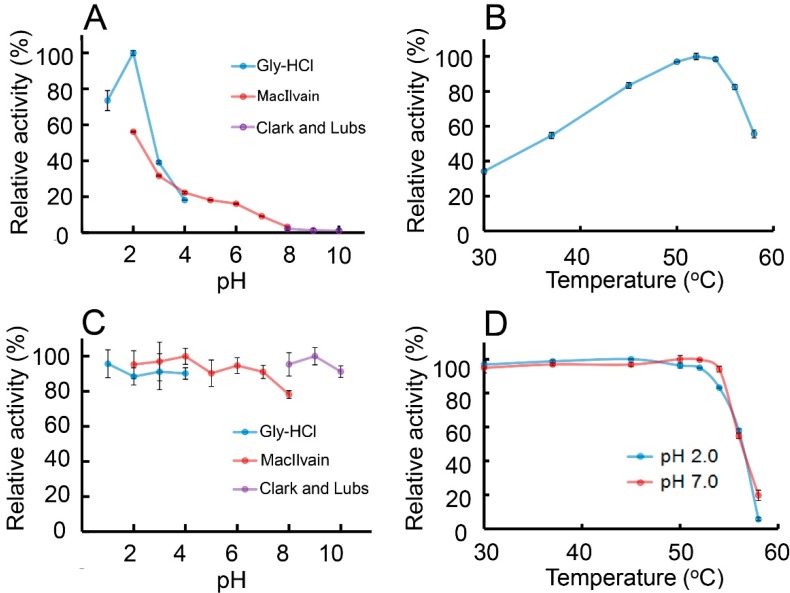
Characterization of the chitinolytic activity of *E. coli*-expressed Protein A-CatD-V5-His. (**A**) pH profile; (**B**) temperature profile; (**C**) pH stability profile and (**D**) thermostability profile of the chitinase activity for recombinant CatD were measured as described in Experimental Section. The results are presented as percentage of the maximum activity obtained in each series of experiments. Error bars represent the mean ± standard deviation from a single experiment conducted in triplicate.

To further characterize *E. coli*-expressed CatD of AMCase, we first evaluated the optimal pH for Protein A-CatD-V5-His activity against the chromogenic substrate at 37 °C for 30 min from pH 1.0 to 10.0. As shown in Figure 3A, the highest chitinolytic activity was achieved at around pH 2.0 and was decreasing in less acidic environments (pH 3.0~7.0) (Figure 3A). Chitinolytic activity at pH 2.0 was higher when using Gly-HCl buffer as compared to McIlvaine buffer (Figure 3A).

The effect of temperature on enzyme activity was determined in 0.1 M Gly-HCl buffer at pH 2.0 at temperatures ranging from 30 to 58 °C using 4-nitrophenyl *N,N′*-diacetyl-β-d-chitobioside for 15 min. The rate of the recombinant CatD-catalyzed reaction increased with the rising temperature, reached the maximum level at 54 °C, and then abruptly declined (Figure 3B).

To determine the pH stability of Protein A-CatD-V5-His, the protein was pre-incubated on ice for 60 min at various pH levels in three different buffers (see the Experimental Section) followed by analysis of the enzymatic activity at 37 °C and pH 2.0. The recombinant CatD was stable in both acidic and basic conditions (between 1.0 and 10.0) during the pre-incubation on ice (Figure 3C).

We next assessed the thermal stability of the Protein A-CatD-V5-His by measuring the chitinolytic activity at pH 2.0 (optimal pH) or pH 7.0 (physiological pH). Samples were pre-incubated at the indicated pH for 20 min from 30 to 58 °C. After pre-incubation, we measured the residual activity against the chromogenic substrate at pH 2.0. Recombinant CatD was heat-stable up to 54 °C, at both pH 2.0 and 7.0 (Figure 3D) while its activity decreased at temperatures above 56 °C.

Thus, the enzymatic properties of the recombinant CatD against 4-nitrophenyl *N,N′*-diacetyl-β-d-chitobioside were basically identical with those of the full-length AMCase with regard to the pH and temperature optima as well as pH and thermal stabilities [24].

### 2.5. Protein A-CatD-V5-His Facilitates Chitin Binding

**Figure 4:**
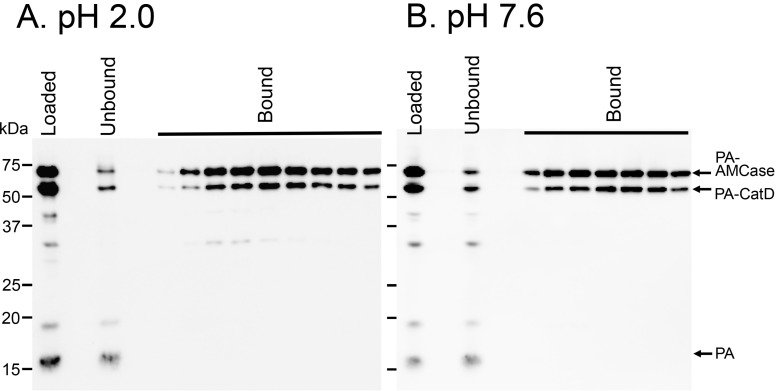
Affinity of Protein A-CatD-V5-His to chitin beads. Protein A-AMCase-V5-His, Protein A-CatD-V5-His and Protein A-V5-His were mixed and loaded onto chitin bead columns. Chitin binding assays were performed at pH 2.0 (**A**) or pH 7.6 (**B**) as described in the Experimental Section. The bound and unbound fractions were analyzed by Western blot using anti-V5-HRP antibody.

The full-length AMCase contains a CBD at the C-terminus of AMCase, whereas it is deleted in CatD (Figure 1A,B). To determine whether Protein A-CatD-V5-His can recognize and interact with chitin, we carried out a binding assay using a chitin-bead column. We mixed Protein A-AMCase-V5-His, Protein A-CatD-V5-His and Protein A-V5-His at pH 2.0 or 7.6 and loaded the samples onto a single chitin beads column equilibrated at the pH conditions described in the Experimental Section. Proteins capable of binding to chitin beads were eluted from the column with 8 M urea [9]. Most of the loaded Protein A-CatD-V5-His and Protein A-AMCase-V5-His at pH 2.0 was detected in the chitin beads-bound fractions, whereas Protein A-V5-His was detected only in the flow-through fraction (Figure 4A). Similar results were obtained with the chitin beads column at pH 7.6 (Figure 4B). These data indicated that the recombinant CatD lacking CBD can bind to chitin with a similar affinity as the full-length AMCase containing CBD at both pH 2.0 and 7.6.

### 2.6. CatD Promotes Chitin Degradation

**Figure 5:**
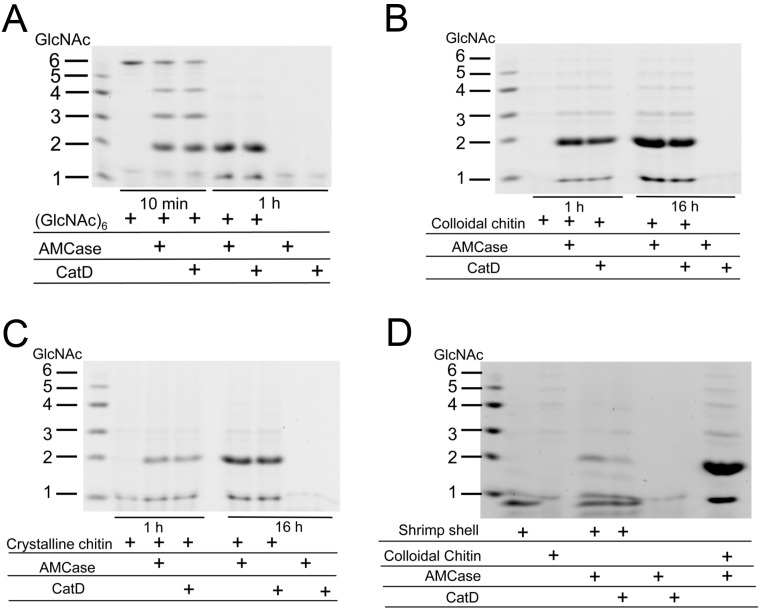
Degradation products of GlcNAc hexamer, colloidal and crystalline chitin and shrimp shell by CatD. GlcNAc hexamer (**A**); colloidal (**B**) and crystalline (**C**) chitin and shrimp shell (**D**) were used as a substrate to determine the chitinase activity of *E. coli*-expressed proteins in 0.1 M Gly-HCl (pH 2.0). Reactions were conducted for 10 min, 1 or 16 h at 37 °C. Shrimp shell (**D**) was digested for 16 h. The chitin fragments generated by the recombinant proteins were analyzed by fluorophore-assisted carbohydrate electrophoresis [9,26]. Chitin oligomers are shown in the left margin as standards. Fluorophore-assisted carbohydrate electrophoresis analysis revealed that Protein A-CatD-V5-His releases primarily *N,N'*-diacetylchitobiose fragments from chitin.

To analyze the effect of the *E. coli*-expressed recombinant proteins, we incubated GlcNAc hexamer and colloidal chitin with Protein A-AMCase-V5-His and Protein A-CatD-V5-His. Mono- and oligosaccharides produced in the reaction were covalently labeled at the reducing end groups with a fluorophore. The resulting fluorescent derivatives were then separated by high-resolution PAGE, as described previously [9,24,26]. We found that both proteins degraded the substrates generating primarily *N,N'*-diacetylchitobiose fragments at pH 2.0 at a similar rate (Figure 5A,B). Protein A-CatD-V5-His also degraded crystalline chitin and shrimp shells at pH 2.0, although its hydrolyzing activity was slightly lower than that of Protein A-AMCase-V5-His (Figure 5C,D). Thus, the CatD of mouse AMCase can recognize high molecular weight chitin substrates and degrade them even in the absence of CBD.

Taken together, these results indicate that *E. coli*-expressed CatD can be considered as a functional enzyme capable of recognizing and digesting oligo- and polymeric chitin.

## 3. Discussion

AMCase has been shown to play important roles in asthma, immune responses and food processing [9,11,12,13,16,22,23]. Little is known, however, about the pathophysiological functions of AMCase in mice and humans. Mouse AMCase contains an *N*-terminal CatD and a *C*-terminal CBD [9]. Human Chit1, which is 52% identical and 60% similar to mouse AMCase [9] also consists of *N*-terminal CatD and *C*-terminal CBD [7,8]. Tjoelker *et al.* expressed and characterized the CatD and CBD of human Chit1 in COS-1 cells and narrowed the CBD region using a biochemical approach [25]. Based on these results, we conducted a homology search and predicted the CatD and CBD of mouse AMCase. It has been suggested that CBD recognizes chitin and CatD mediates its degradation [25]. In this study, we expressed the CatD of mouse AMCase as a recombinant fusion protein of Protein A and V5-His using pEZZ18 vector and examined its properties.

We used pEZZ18 vector [27], which is a Protein A gene fusion vector system based on two synthetic IgG-binding domains (ZZ) of *Staphylococcus aureus* Protein A. This vector has been used for the extracellular expression of secretory proteins [27,28,29,30]. Using this vector, we have established an *E. coli* expression system that allows for the periplasmic production of an active AMCase as a fusion protein with Protein A and V5-His [24]. Furthermore, using pEZZ18, we succeeded in expressing glucoamylase of *Caulobacter crescentus* CB15, which formed inclusion bodies in the pET system, in a soluble and active form in *E. coli* [31]. Thus, Protein A aids in folding and solubilizing the fused protein moiety.

The soluble recombinant product can be recovered in a one-step procedure by IgG affinity chromatography. The bound protein could be eluted with 0.1 M Gly-HCl (pH 2.5). This method can only be used if the fusion product is stable under these conditions. The CatD showed acid stability at pH 1 to 3 (Figure 3C). Thus, we could use IgG Sepharose as an affinity chromatography resin for the purification of Protein A-CatD fusion protein.

The objective of the studies described here was to compare the enzymatic properties of the full-length AMCase and its CatD expressed in *E. coli* cells. We have reported that *E. coli*-expressed AMCase showed properties similar to those of the native enzyme from mice [9] or CHO-expressed AMCase [24]. Here, we show that the chitinolytic activity of the recombinant CatD against 4-nitrophenyl *N,N′*-diacetyl-β-d-chitobioside was equivalent and comparable to the *E. coli*-produced full-length AMCase as for the specific activity, pH and temperature optima as well as pH and thermal stabilities. Furthermore, the CatD degraded colloidal chitin and produced primarily *N,N'*-diacetylchitobiose. These results indicate that the *E. coli*-expressed Protein A-AMCase-V5-His and Protein A-CatD-V5-His are equivalent under our experimental conditions. Our results clearly indicate that the primary structure of CatD is sufficient to form a proper tertiary structure for chitinolytic activity in the absence of CBD.

The recombinant CatD of AMCase bound to chitin beads and cleaved the GlcNAc hexamer, colloidal and crystalline chitin and shrimp shell at pH 2.0. These results indicate that the CatD of mouse AMCase can recognize high molecular weight chitin substrates and degrade them in the absence of CBD. A high-resolution analysis by X-ray crystallography shows that a long cleft, which is lined with aromatic residues, is the site of chitin binding [32]. These aromatic residues are highly conserved across species [5]. Thus, the CatD binds with chitin beads probably using the aromatic residues, which can be involved in chitin recognition and binding.

The CatD showed reduced hydrolytic activity towards crystalline chitin and shrimp shell, when compared with the full-length AMCase. *Serratia marcescens* 2170 produced chitinase C1 and C2 [33]. Suzuki *et al.* reported that chitinase C1 is the full-length chitinase consisting of CatD and CBD, whereas chitinase C2 contains CatD and is generated by proteolytic removal of CBD from chitinase C1 [34]. These authors showed that chitinase C2 possessed chitin binding activity and degraded chitin substrates, although chitinase C2 showed reduced hydrolytic activity towards powdered chitin but not towards colloidal chitin when compared with chitinase C1. Our present data concerning the CatD of AMCase are essentially consistent with chitinase C2 with regard to the binding and degradation of chitin. Thus, these results illustrate the importance of the CBD of chitinases for the efficient hydrolysis of crystalline chitin and shrimp shells.

We recently reported that AMCase mRNA is synthesized at extraordinarily high levels in the mouse stomach [22,23,35]. The stomach is an important organ that plays fundamental roles in the digestion of foods and the protection against harmful organisms. In the mouse stomach, a significant amount of pepsin, the protein-degrading enzyme of the digestive system [36,37], is produced and secreted [22]. It is possible that AMCase is cleaved by pepsin to yield CatD and CBD in the hinge region. This notion is worth considering because Chit1 was reported to be cleaved into CatD and CBD in human macrophage [38]. Recombinant mouse AMCase and CatD are most active at pH 2.0, which reflects the stomach’s acidity and shows marked acid stability (Figure 3A,C). Furthermore, both the full-length AMCase and CatD can recognize various chitin substrates and degrade them. The unusual acid dependence and stability of the mouse AMCase CatD in acidic conditions allow for the efficient digestion of chitinous materials under the extreme acidic environment in the stomach. We plan careful analysis to examine whether AMCase can function as a digestive enzyme that breaks down chitin-containing materials in the mouse stomach.

AMCase is potentially an important downstream effector of interleukin-13 stimulation in Th2 helper cell-mediated immune responses to pathogens, parasites and ovalbumin. Elevated AMCase levels in lungs or eye may lead to diseases such asthma, allergic inflammation, ocular allergy, and dry eye syndrome (reviewed in [19]). Inhibition of AMCase has been suggested as an important therapeutic target in these diseases [11,14,15]. Since mammals synthesize two chitinases, Chit1 and AMCase, development of inhibitors that are selective for AMCase is needed. The recombinant mouse AMCase and its CatD possessed enzymatic properties very similar to mammalian cell-produced recombinant protein. Our recombinant proteins can be used for understanding the pathophysiological roles of AMCase in disease conditions potentially leading to further development of specific AMCase inhibitors.

## 4. Experimental Section

### 4.1. E. coli Expression Vector

To express the CatD of mouse AMCase as a recombinant fusion protein with Protein A and V5-His, the CatD region was amplified from the full-length mouse AMCase expressing plasmid DNA (the pEZZ18/AMCase-V5-His) using KOD Plus DNA polymerase (Toyobo, Osaka, Japan) and oligonucleotide primers (Sigma–Aldrich Life Science Japan, Ishikari, Japan) anchored with the restriction sites EcoRI and XhoI as described previously [24]. The forward primer (5'-**CATG**GAATTCGTACAATCTGATATGCTATTTCACC-3’) for CatD contains a 6 bases-long EcoRI recognition sequence (underlined) and a 25 bases-long AMCase sequence corresponding to nucleotides 80~103 of the AMCase cDNA (GenBank accession number AK160173.1). We designed the reverse primer, which is in frame with the *C*-terminal region of V5-His. The reverse primer (5'-**GTGAC**CTCGAGCACTCCCACTTCCTGGAGGAGTAG-3') contains the XhoI recognition sequence (underlined) and is complementary to nucleotides 1230~1253 of the AMCase cDNA. Both primers contain the 4~5 bases-long extra nucleotides (boldfaced) to efficiently cleave close to the end of the amplified cDNAs by a restriction enzyme. The amplified DNA contains one EcoRI and one XhoI sites anchored with the PCR primers. The PCR product was purified using the Wizard SV Gel and PCR Clean-Up System (Promega, Madison, WI, USA) and then digested with EcoRI and XhoI. The DNA fragment was purified using 1.5% agarose gel electrophoresis and the Clean-Up System and was finally subcloned into a similarly digested pEZZ18 expression vector. The entire nucleotide sequence of the resulting plasmid DNA (pEZZ18/CatD/V5-His) was confirmed by sequencing (Eurofins Genomics, Ebersberg, Germany).

### 4.2. Preparation of the Recombinant CatD Expressed in E. coli

Transformed *E. coli* BL21 (DE3) strains were grown in 1 L LB medium containing 100 µg/mL ampicillin at 37 °C for 18 h. Cells were harvested by centrifugation at 5000× *g* for 20 min at 4 °C. The recombinant CatD was prepared from the periplasmic fraction of the *E. coli* and purified by IgG Sepharose chromatography, followed by Ni Sepharose chromatography as described previously [24]. The protein-containing fractions were desalted using PD MidiTrap G-25 (GE Healthcare, Little Chalfont, UK) equilibrated with the TS buffer (20 mM Tris-HCl (pH 7.6), 150 mM NaCl and protease inhibitor).

We also produced Protein A-AMCase-V5-His and Protein A-V5-His as described previously [24].

### 4.3. SDS-Polyacrylamide Gel Electrophoresis and Western Blotting

The obtained protein fractions were analyzed using standard SDS-polyacrylamide gel electrophoresis (PAGE), followed by Western blotting using anti-V5-HRP monoclonal antibody (Invitrogen, Carlsbad, CA, USA). We used All Blue molecular weight marker (All Blue, Bio-Rad Laboratories, Hercules, CA, USA).

### 4.4. Chitinase Enzymatic Assays

Chitinolytic activity was determined using the synthetic chromogenic substrate, 4-nitrophenyl *N,N′*-diacetyl-β-d-chitobioside (Sigma–Aldrich), at a concentration of 200 μM. Each reaction was performed in triplicate. All enzymatic reactions for the determination of optimum pH and temperature were conducted in a volume of 50 μL containing *E. coli*-expressed protein in 0.1 M Gly-HCl buffer or McIlvaine’s buffer. Reactions performed for the optimum pH and kinetic assays were conducted for 30 min at 37 °C. The reactions were halted with the addition of 20 μL of 1 M sodium carbonate to the reaction mixture. The absorbance of the liberated 4-nitrophenolate ion was measured at 405 nm.

### 4.5. Chitin Binding Assay

Protein A-AMCase-V5-His, Protein A-CatD-V5-His and Protein A-V5-His (each 20 µg) were mixed after adjusting the binding buffers’ pH values to pH 2.0 or 7.6 in 0.5 M NaCl. The mixed solutions were loaded onto a chitin bead (500 µL, New England Biolabs, Ipswich, MA, USA) column equilibrated with the binding buffers (0.5 M NaCl in 100 mM Gly-HCl (pH 2.0) or 0.5 M NaCl in 20 mM Tris-HCl (pH 7.6)) and recirculated ten times to bind the recombinant proteins to the chitin beads. The flow-through fractions were pooled as an unbound fraction. The columns were washed with 10-column volumes of the same buffer and then washed with 5-column volumes of 2 M NaCl in 100 mM Gly-HCl (pH 2.0) or 2 M NaCl in 20 mM Tris–HCl (pH 7.6), respectively. Bound proteins were eluted with 8 M Urea and 20 mM Tris–HCl (pH 7.6). The bound and unbound fractions were analyzed by Western blot using an anti-V5-HRP monoclonal antibody.

### 4.6. Degradation of GlcNAc Hexamer, Colloidal and Crystalline Chitin and Shrimp Shell by AMCase and CatD

Colloidal chitin was prepared from shrimp shell chitin (Sigma-Aldrich), as described previously, and used as a substrate to determine the chitinase activity [9]. Shrimp shell chitin was powdered in a Wiley mill (Thomas Scientific, Swedesboro, NJ, USA) to 250 μm particle size. We used this preparation as crystalline chitin. Dried shrimp shell (Tosayasyoten, Tokyo, Japan) was powdered using a mortar. All enzymatic reactions using colloidal chitin (at a final concentration of 1 mg/mL), crystalline chitin (1 mg/reaction) or dried shrimp shell (1 mg/reaction) as substrates were carried out in a volume of 50 μL containing *E. coli*-expressed proteins in 0.1 M Gly-HCl buffer (pH 2.0). The reactions were incubated for 10 min, 1 h or 16 h at 37 °C. The chitin fragments generated from these reactions were labeled covalently at their reducing end groups with the fluorophore 8-aminonaphthalene-1,3,6-trisulphonic acid (ANTS, Invitrogen), and the resulting fluorescent derivatives were separated by high-resolution PAGE, as described by Jackson [26]. *N*-acetyl chitooligosaccharides (Seikagaku Corporation, Tokyo, Japan) were used as standards.

## 5. Conclusions

Our results indicate that the *E. coli*-expressed Protein A-AMCase-V5-His and Protein A-CatD-V5-His are equivalent under our experimental conditions and that the primary structure of CatD is sufficient to form a proper tertiary structure for chitinolytic activity in the absence of CBD. Thus, the CatD of mouse AMCase can recognize chitin substrates and degrade them in the absence of CBD. Our results suggest that the development of potentially druggable AMCase-specific inhibitors should focus on compounds binding to CatD enabling a more potent chitinolytic activity inhibition of AMCase.

## Supplementary Materials

Supplementary materials can be found at http://www.mdpi.com/1422-0067/16/02/4028/s1.
